# What is the scope of improving immigrant and ethnic minority healthcare using community navigators: A systematic scoping review

**DOI:** 10.1186/s12939-016-0298-8

**Published:** 2016-01-15

**Authors:** Nusrat Sharmeen Shommu, Salim Ahmed, Nahid Rumana, Gary R. S. Barron, Kerry Alison McBrien, Tanvir Chowdhury Turin

**Affiliations:** Department of Family Medicine, Room G012F, Health Sciences Center, University of Calgary, 3330 Hospital Drive Northwest, Calgary, AB T2N 4N1 Canada; Sleep Center, Foothills Medical Center, University of Calgary, 1403 29 Street NW, Calgary, AB Canada; Department of Community Health Sciences, University of Calgary, 3280 Hospital Drive Northwest, Calgary, AB T2N 4Z6 Canada

**Keywords:** Immigrant, Healthcare, Navigator, Primary care

## Abstract

**Introduction:**

Immigrants are among the most vulnerable population groups in North America; they face multidimensional hurdles to obtain proper healthcare. Such barriers result in increased risk of developing acute and chronic conditions. Subsequently a great deal of burden is placed on the healthcare system. Community navigator programs are designed to provide culturally sensitive guidance to vulnerable populations in order to overcome barriers to accessing healthcare. Navigators are healthcare workers who support patients to obtain appropriate healthcare. This scoping review systematically searches and summarizes the literature on community navigators to help immigrant and ethnic minority groups in Canada and the United States overcome barriers to healthcare.

**Methods:**

We systematically searched electronic databases for primary articles and grey literature. Study selection was performed following the preferred reporting items for systematic reviews and meta-analyses (PRISMA) statement. Articles were selected based on four criteria: (1) the study population was comprised of immigrants or ethnic minorities living in Canada or the United States ; (2) study outcomes were related to chronic disease management or primary care access; (3) the study reported effects of community navigator intervention; (4) the study was published in English. Relevant information from the articles was extracted and reported in the review.

**Result:**

Only one study was found in the literature that focused on navigators for immigrants in Canada. In contrast, 29 articles were found that reported navigator intervention programs for immigrant minorities in the United States. In these studies navigators trained and guided members of several ethnic communities for chronic disease prevention and management, to undertake cancer screening as well as accessing primary healthcare. The studies reported substantial improvement in the immigrant and ethnic minority health outcomes in the United States. The single Canadian study also reported positive outcome of navigators among immigrant women.

**Conclusion:**

Navigator interventions have not been fully explored in Canada, where as, there have been many studies in the United States and these demonstrated significant improvements in immigrant health outcomes. With many immigrants arriving in Canada each year, community navigators may provide a solution to reduce the existing healthcare barriers and support better health outcomes for new comers.

## Introduction

Community navigators are trained, culturally perceptive healthcare workers who serve as a link between patients and healthcare providers in order to reduce healthcare disparities [1–3]. They may also be referred to as patient navigators, community health workers, outreach workers, promotoras, lay health educators, health advocates, peer counselors or medical assistants [4]. Unlike physicians and nurses, community navigators do not provide healthcare services directly; they offer culturally tailored educational support to patients, aid communication between patients and physicians, and guide patients in overcoming barriers to obtaining appropriate healthcare [1, 5]. The first community navigator program was developed in New York City, USA to serve African American women with breast cancer in 1990 [6]. In Canada, the navigation program was first adopted in the cancer care system of Nova Scotia in 2001 [7].

Community navigators were created to provide appropriate support to underserved populations who have the least ability to access healthcare [4]. A significant part of this vulnerable patient group is comprised of immigrants and ethnic minorities, who are faced with numerous barriers to care [4] in the community in which they reside. These include language barriers, low levels of health literacy, financial issues, unfamiliarity with the healthcare system, as well as cultural and religious discordance [8]. People migrate to North America from different cultural and religious backgrounds that play a vital role in their attitude towards healthcare. Healthcare providers often fail to understand and respect the cultural aspects of the immigrant patients, which may drive them away from seeking primary healthcare [9]. Language barriers between the patient and the physician often result in unsuccessful communication where the physician does not understand the patient’s needs and thus fails to provide proper service [9]. Moreover, many immigrants, especially during the early stage of settlement face major socioeconomic obstacles that can be a major limitation in accessing primary care [1]. The healthcare systems prevailing in North America are often different from the native countries of immigrants, making it difficult for them to navigate through the system. This difference in systems can be a major barrier for immigrants to access healthcare [9]. Without adequate access to care, including comprehensive primary healthcare, minority groups remain at higher risk of developing acute and chronic illnesses, including diabetes, hypertension, coronary disease and cancer [4, 10, 11]. As a result, they are more likely to present with advanced disease, which ultimately places added pressure on the healthcare system.

Community navigators are particularly relevant for Canada [12] and the United States [13], which accommodate many immigrants [14]. Although community navigator programs were initially introduced to ease disparities in cancer care among vulnerable populations [15], the United States is now widely using community navigators to address other healthcare gaps [4]. Canadian navigation programs are still focused on cancer treatment and care, mostly among the general population, rather than specifically among the underserved immigrant group [6]. However, recently, a few studies reported immigrant oriented cancer navigator programs in Toronto and Vancouver [6]. But the use of community navigators targeting different non-cancer health disparities among immigrant minorities has not yet been widely adopted in Canada.

Although Canada has a universal healthcare system, there is ample evidence documenting that vulnerable populations including immigrants and refugees experience substantial barriers to accessing healthcare, including primary care [9, 16, 17]. In this scoping review we summarize the literature regarding the use of community navigators for supporting immigrant populations and ethnic minorities for chronic disease management in primary care settings in the United States and Canada. We have also pointed out the lack of evidence for involving navigators to support Canadian immigrant populations and draw attention to the opportunity to undertake navigator programs to address health disparities in Canada.

## Method

### Literature sources and search strategy

**Fig. 1:**
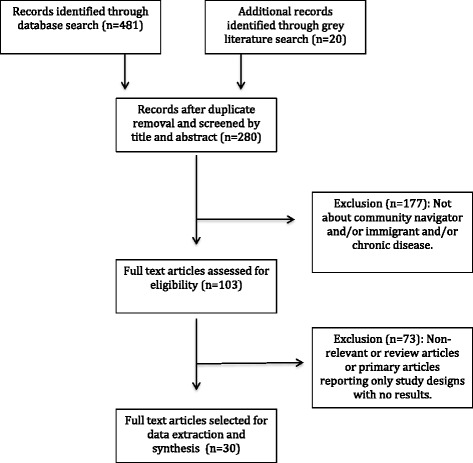
Flow diagram describing the systematic literature search for studies examining the effect of community navigator in improving chronic disease management and primary care adoption by immigrant and ethnic minorities in North America

We systematically searched the following electronic databases: Medline, EMBASE, EBM Reviews (Cochrane), PsycINFO, PubMed, PubMed Central, Scopus, Web of Science, CINAHL, and Academic Search Complete for studies that employed community navigators to address healthcare disparities among immigrants and ethnic minority populations in North America. Additionally, we searched OpenDOAR, Health Sciences Online (HSO), Turning Research into Practice (TRIP), OAISter (WorldCat), Canadian Institute for Health Information (CIHI), Public Health Agency of Canada (PHAC), Health Canada and National Institutes of Health (NIH) for grey literature. Medical Subject Heading (MeSH) terms and keywords for community navigators (promotora/patient navigator/patient liaison/patient advocate/lay health worker/community health worker/community navigator/patient facilitator/health educator/case manager/outreach worker) were combined with those for chronic disease or primary care (Chronic disease/chronic illness/chronic condition/cardiovascular disease/cerebrovascular disorders/hypertension/myocardial infarction/heart failure/coronary disease/heart diseases/stroke/diabetes/pulmonary diseases/chronic obstructive pulmonary disease/asthma/chronic kidney disease/carcinoma/neoplasms/primary care/primary healthcare/general practitioner/family medicine/family doctor/family physician/walk-in clinic), immigrant or ethnic minority population (Immigrant/ emigrant/ alien/foreigner/minority group/visible minority/vulnerable minority/migrant/ethnicity/ethnic group/Latino/Hispanic/ African/Black/South Asian/Asian/Vietnamese/Chinese/Korean/Filipino/Vietnamese/East Indian/Arab/Middle East/Caribbean) and North America (Canada/USA/United States/America/ US/North America). We conducted this scoping review according to the methods described by Arksey and O’Malley [18] and the search process is shown in the flowchart presented in Fig. 1 [19].

### Study selection

We included primary studies that met the following criteria: (1) the study population was comprised of immigrants or ethnic minorities living in Canada or the United States ; (2) study outcomes were related to chronic disease management or primary care access; (3) the study reported effects of community navigator intervention either qualitatively and/or quantitatively; (4) the study was published in English. Conference abstracts, poster summaries and dissertation reports were considered eligible for inclusion in order to ensure incorporation of all relevant studies. We excluded articles that reported the study design only but no outcomes. Review articles were also excluded.

### Data extraction

For each study, we extracted the following: information regarding study design and period, target population along with size (n) and age, clinical characteristics of the study population, primary outcomes and major effects of the community navigator intervention. The major focuses of data extraction were cardiovascular disease, diabetes and other chronic disease management and acceptance of primary care in the vulnerable ethnic population under study.

## Search results

Our initial search resulted in 501 potentially relevant peer-reviewed abstracts and grey literature papers (Fig. 1). After elimination of 221 duplicates, we excluded an additional 177 articles during screening based on the title and abstract, then assessed the full text of the remaining 103 articles. A total of 30 primary studies (including one PhD dissertation report) fulfilled our inclusion criteria and were selected for the scoping review. The PhD thesis report was the only study done in Canada, the rest of the studies were done in the United States. Most of the studies in the United States were in Latino and African American communities. Four additional studies focused on South Asian, Filipino, Korean and Vietnamese communities. Study duration ranged from 3 weeks to 88 months and sample size (n) from 39 to 849 participants (Table 1). With the exception of the thesis dissertation, which was a case study, all the other studies used a pre-test/post-test assessment of the population for a community navigator intervention, among them 16 were controlled trials and rest of the remaining 13 were single arm intervention studies (Table 1).

**Table 1:** Effect of community navigator intervention to improve chronic disease management and participation in primary care for cancer screening among immigrant and ethnic populations in the United States

Study	Immigrant Ethnic Population	Size (n)	Age (y)	Study period	Clinical Condition/Risk Factor	Primary Outcome	Major Effects of Intervention on Primary Outcome
Islam et al. [23]	Sikh, Asian Indian	108	18-75	6 months	Type 2 diabetes	BMI, weight, glucose level, blood pressure,total cholesterol.	Significant reduction in glucose level (22.4 %), weight (2.99 %) and BMI (2.88 %) and blood pressure (systolic 10.18 %, diastolic 6.14 %), all Ps < 0.01. No significant reduction in cholesterol.
Islam et al. [34]	Korean American	48	18-75	6 months	Type 2 diabetes	BMI, weight, glucose level, blood pressure, total cholesterol.	Positive directional changes for the treatment group, though none were statistically significant at *P* < 0.05
Lujan et al. [22]	Hispanic	150	Mean age 50	6 months	Type 2 diabetes	Blood HbA1c level (%)	Significant reduction by 0.45 % (*P* < 0.001)
Corkery et al. [32]	Hispanic	40	NR	NR	Type 2 diabetes	Blood HbA1c level (%)	Significant reduction by 1.8 % (*P* < 0.004)
Spencer et al. [30]	African American, Latino	164	≥18	6 months	Type 2 diabetes	Blood HbA1c level (%)	Significant reduction by 0.8 % (*P* < 0.01)
Fedder et al. [27]	African-American	117	Mean age 57 ± 12	28 months	Type 2 diabetes	Emergency Room (ER) visit, hospitalization	ER visits and hospitalization declined by 40 % and 33 % respectively (*P* = 0.02)
Palmas et al. [33]	Hispanic	360	35-70	12 months	Type 2 diabetes	Blood HbA1c level (%)	No significant improvement on HbA1c level
Rothschild et al. [35]	Hispanic	144	≥18	2 years	Type 2 diabetes	Blood HbA1c level (%) and blood pressure	Significant between group differences in blood HbA1c level at the end of year 1 (−0.55 *P* = .021) and year 2 (−0.69 *P* = .005). No significant reduction in blood pressure.
Ingram et al. [37]	Hispanic	70	Average age 60	12 months	Type 2 diabetes	Blood HbA1c level (%)	Significant reduction by 1% (*P* = 0.01)
Perez-Escamilla R et al. [36]	Latino	211	≥21	18 months	Type 2 diabetes	Blood HbA1c level (%)	Significant group difference in reduction of HbA1c level (−0.55, *P* = 0.009)
Prezio et al. [31]	Hispanic	189	18-75	12 months	Type 2 diabetes	Blood HbA1c level (%)	Significant reduction by 0.7 % (*P* = 0.02)
Ursua et al. [43]	Filipino	39	25-75	4 months	Hypertension management	Blood pressure control, appointment keeping and medication adherence	27.3 % increase in number of individuals with controlled blood pressure
Hurtado et al. [4]	African American, American Indian, Hispanic and Filipino	849	Mean age 48	3 years	Cardiovascular risk factors	Heart healthy knowledge, heart healthy meal, CVD risk factor behavior	Heart healthy knowledge score and CVD risk factor behavior increased by 26 % and 44 % (*P* < 0.001) respectively.
Sanchez et al. [41]	Latino	96	≥18	9 weeks	Hypertension management	Food habit and physical activity	Significant improvement in self reported behavior
Balcazar et al. [21]	Latino	320	NR	6 months	Cardiovascular risk factors	Health habits, community referrals, screening, information sharing	18 % improvement of average overall score (*P* < 0.001)
Spinner et al. [40]	Latino	435	NR	65 days	Cardiovascular risk factors	Physical activity, heart healthy knowledge, heart healthy meal	Significant increase in physical activity (21 %), heart health knowledge (27 %) and heart healthy meal preparation (15 %), all Ps < 0.001.
Balcazar et al. [26]	Hispanic	85	NR	12 months	Cardiovascular risk factors	Weight, BMI, blood pressure, LDL, HDL, triglyceride level, HbA1c	Significant reduction in LDL cholesterol (*P* < 0.001), triglyceride (*P* = 0.02) levels.
de Heer et al. [39]	Hispanic	328	30-75	4 months	Cardiovascular risk factors	Protective health behaviors, health beliefs, contextual and social factors	Improved nutritional consumption
Balcazar et al. [38]	Hispanic	98	52.3	9 weeks	Hypertension	Weight, BMI, blood pressure, food habit	Significant reduction in salt, sodium and fat intake
Koniak-Griffin et al. [24]	Latino women	223	35-64	32 months	Cardiovascular risk factors	Weight, BMI, blood pressure, lipids, blood glucose, food physical exercise	Significant improvements in dietary habits, waist circumference and physical exercise.
Kandula et al. [42]	South Asian	63	30-59	6 months	Cardiovascular risk factor	Weight, blood pressure, cholesterol, HbA1c level, health behaviors, knowledge, coping, and exercise confidence	Significant between group differences in weight (−3.2 lb, *P* = 0.04) and HbA1c (−0.43 %, *P* < 0.01) reductions.
Hunter et al. [44]	Hispanic	101	40-70	28 months	Chronic disease prevention	Participation in routine preventive chronic disease screening	Women in the intervention group were 35 % more likely to go for rescreening than those in the control group.
Staten et al. [10]	Hispanic	254	≥18	41 months	Chronic disease prevention	BMI, waist circumference, blood pressure, blood glucose, cholesterol, triglycerides, dietary habits and physical activity	Significant reduction in BMI (*P* = 0.04), waist circumference (*P* < 0.001), blood pressure (*P* < 0.001) and total cholesterol (*P* = 0.008)
Schwartz et al. [28]	Hispanic	450	18-84	3 years	Obesity and metabolic syndrome	BMI, waist circumference, blood pressure, cholesterol, glucose, HbA1c, dietary habits, physical activity.	72 %, 69 %, 59 %, and 48 % of participants reduced weight, BMI, waist circumference and blood pressure respectively. Glucose, HbA1c and total cholesterol decreased by 6.3 %, 3.8 % and 2.3 % respectively.
Martin et al. [25]	African-American	42	21-50	6 months	Asthma	Asthma self efficacy	Significant improvement in asthma quality of life (*P* = 0.002) and coping (*P* = 0.01)
Allen et al. [46]	Latina	155	≥18	6 months	Breast, cervical and colorectal cancer screening	Adherence with screening recommendation	24 % and 8 % increase in adherence with breast cancer and to all cancer screening recommendations for one’s age respectively, however, these changes were not statistically significant.
Livaudais et al. [47]	Hispanic women	70	40-79	6 months	Breast cancer screening	General Cancer knowledge, screening practices and intention to be screened	Significant improvement in knowledge on cancer prevention (26 %, *P* = 0.001), intention to do mammogram (8 %, *P* = 0.014) and discussing mammograms with doctor (30 %, *P* < 0.001)
Percac-Lima et al. [29]	Latina	786	22-86	88 months	Cervical cancer screening	Missing colposcopy appointment, time to colposcopy and severity of cervical pathology	Significant reduction in missing colposcopy (4.1 %, *P* = 0.024) and severity of cervical pathology (9.9 %, *P* = 0.035)
Chen et al. [11]	African Americans and Hispanics	532	≥50	31 months	Colon Cancer screening	Colonoscopy completion, endoscopic findings, and patient satisfaction about navigator	66 % completed colposcopy screening, 16 % had adenomas, only 5 % had inadequate bowel prep, 66 % patients agreed that they would not do colonoscopy without navigation.

In general, the articles evaluated the effectiveness of community navigators in increasing capacity among immigrants and ethnic minorities for chronic disease self-management, prevention, and, when needed, accessing primary care. The Canadian case study evaluated the effectiveness of community navigators in improving health disparity among immigrant women. Among the United States based studies, 11 studied the effectiveness of community navigators in management of type 2 diabetes, 10 examined cardiovascular disease management including hypertension, two studied chronic disease prevention in general, one examined asthma self-efficacy, one considered metabolic syndrome, and four evaluated the participation in cancer screening in primary care. Table 1 summarizes the outcomes of the studies.

## Literature summary

### Community navigators in addressing health equity for immigrant and refugee women in Canada

Only one study (a doctoral thesis) was found to meet the inclusion criteria that assessed the role of community navigators in Canada [20]. The author investigated the role of the Multicultural Health Brokers Co-operative (MCHB Co-op) in addressing health disparities of immigrant women and their families in Edmonton through interviews, direct observation, analysis of documents, and analysis of the client database. MCHB Co-op is a multicultural health worker co-operative, which provides linguistically and culturally tailored assistance to immigrant and refugee women and their families in collaboration with health and social service providers. The organization uses a holistic approach to assist immigrant and refugee women from approximately 80 different countries by implementing several health and non-health programs. These programs include perinatal healthcare, home visitation for infants, multicultural family connection, childcare services and support for children with disability, youth mental health and leadership, a seniors program, and new Canadians clinic [20]. The author found that in addition to successfully navigating immigrant and refugee women and their families towards healthcare and social services, MCHBs also facilitated their settlement, adaptation, and integration into Canadian society [20].

### Community navigators to educate immigrant and/or ethnic populations in the United States

Common themes were found among the United States based studies. Community navigators were employed to address health disparities among immigrants and assist them in moving through the healthcare system in the United States. Navigators were selected from the community based on their cultural competence, interpersonal skills and helping attitude towards their community and were given comprehensive training by health professionals. Major roles of the navigators included providing culturally tailored health education, lifestyle workshops, self-care training and guidance to overcome barriers to accessing the healthcare system. The initial training was usually conducted through a series of individual or group sessions and workshops; the patients are monitored afterwards through telephone calls, home visits, support groups, and walking clubs at regular intervals over the trial period. The navigators also distributed educational materials and videos describing healthy diet, exercise, self-monitoring of health risk factors, handling emergency conditions and medication adherence [8, 21].

Both controlled trials and single arm intervention studies were employed to evaluate the effectiveness of community navigators in the United States. In the controlled trials the control groups did not receive any intervention from the community navigators; rather they received usual care, or only educational materials, or alternative education [22–25]. The intervention group received specific health training from community navigators and the language of instruction was the native language of the target population, either solely or in combination with English. Studies were conducted in clinics, primary healthcare settings, community-based organizations, churches, or at social gatherings or a combination of two or more of these settings [26–29].

#### Community navigator in diabetes management

Navigators have been successfully employed in order to reduce the burden of diabetes in the United States. Eight studies examined the effects of patient navigators in improving outcomes for patients with diabetes. All included adult participants belonging to the target community diagnosed with [8, 22, 27] or at risk of [23] type 2 diabetes. In one study, the community navigators were also patients with diabetes (peer navigators) or had a family history of diabetes [8]. After obtaining extensive training on diabetes management from healthcare professionals, bilingual community navigators provided patients with culturally tailored education on healthy lifestyle and diabetes self-management including dietary habits, physical exercise, glucose testing, medications and utility of primary healthcare [27, 30, 31]. Primary outcomes, including HbA1c level, blood pressure, body weight, cholesterol levels, diabetes knowledge and beliefs, healthy eating and physical activity were measured at baseline followed by one or multiple times after the navigator intervention began [22, 32, 33]. Changes in utilization of primary care, emergency room visits and hospital admissions after community navigator intervention were also assessed [23, 27].

To cite a specific example, project RICE (Reaching Immigrants through Community Empowerment) at New York State University investigated the effect of community navigators in diabetes prevention in both the Sikh Asian Indian and Korean communities in New York City [23, 34]. In a non-randomized controlled trial, a total of 126 adults at risk of diabetes from two Sikh communities were enrolled [23]. Participants from one neighborhood were allocated to the community navigator intervention group and those from the other neighborhood to the control group. The control group received standard healthcare as needed. Primary outcomes including weight, BMI, blood pressure, glucose, healthy eating, physical activity and utilization of primary healthcare were measured at baseline and 3 months and 6 months after intervention. All surveys and interviews were administered in Punjabi, the native language of the Sikh community. Between-group differences were observed in terms of blood glucose level, physical activity, dietary habits and diabetes knowledge; the culturally tailored navigator intervention group exhibited greater improvement compared to the control group and differences were statistically significant [23]. Project RICE implemented another pilot study in Korean immigrants in New York, where 48 Korean Americans were randomly distributed among treatment and control groups [34]. Although between group differences in the primary outcomes were not statistically significant, positive directional changes were observed for the treatment group [34].

The other studies in diabetes management did not directly focus on immigrants; rather they targeted ethnic minorities (Hispanic and African-American) and did not distinguish between immigrant and non-immigrant groups. In several studies, culturally designed diabetes education from Spanish-speaking community navigators led to significant reduction of blood HbA1c levels among Hispanic adults with type 2 diabetes [8, 22, 30, 35–37]. The extent of HbA1c level reduction was higher in patients who received more frequent navigator contacts [8]. Participation in community diabetes education had an additional effect of reducing HbA1c levels among African and Hispanic diabetic patients receiving medical care from physicians [31]. Scores for diabetes knowledge and diabetes health belief also increased after navigation [22]. More importantly, total ER visits and ER admissions in Hispanic adults with diabetes were significantly reduced after receiving navigator guidance [27]. These positive outcomes were achieved after a period between 6 months and one year [8, 22, 31]. However, in two of the studies improvements in HbA1c levels, diabetes knowledge and self-care practices were not statistically significant after 1 year of community navigator intervention [32, 33].

#### Community navigator in cardiovascular disease management

Our search found several studies that examined the National Heart, Lung, and Blood Institute (NHLBI) Community Health Worker (CHW) Health Disparities Initiative where trained community navigators educated underserved communities using a standard curriculum known as ‘heart health curriculum’ developed by NHLBI [4, 21]. The ‘heart health curriculum’ is culturally customized and designed to improve health knowledge and lifestyle behaviors in order to reduce cardiovascular risks [4]. It includes education sessions followed by home visits and telephone calls, together with easy to read educational materials and videos. Trained community navigators use of the heart health curriculum led to significant improvements in clinical CVD risk factors (e.g., weight, BMI, blood pressure, LDL, HDL and triglyceride) as well as lifestyle behaviors for healthy heart (e.g., smoking, physical activity, intake of salt and sodium, and cholesterol and fat) among Hispanic men and women [21, 24, 26, 38–41]. Sometimes desirable results were achieved in as little as nine weeks [38]. African American, south Asian, and Filipino participants also demonstrated similar positive outcomes when community navigators educated them using the heart health curriculum [4, 42, 43].

#### Community navigators in metabolic syndrome management

A community navigator-led wellness program was designed to reduce the risks of metabolic syndrome among Hispanic adults [28]. The program focused on improving nutrition and physical activity through increased community support and infrastructure for healthy lifestyle. The study found significant reductions in weight, BMI, waist circumference and blood pressure among participants [28].

#### Community navigators in asthma management

In one study [25] African American adults with poorly controlled persistent asthma were provided disease education specific to controller medications, spacers, inhaler technique, symptom monitoring, communication with providers, asthma triggers, and smoking. Education took place through four group sessions followed by home visits and telephone calls. The participants demonstrated improvements in asthma self-efficacy, asthma quality of life and coping [25].

In addition to guiding vulnerable population in chronic disease self-management, community navigators also aided in chronic disease prevention by increasing their compliance with routine preventative exams [44].

#### Community navigator in improving cancer screening in primary healthcare

Studies on immigrant minority groups in the United States showed that they have lower screening rates for cervical, breast, and colorectal cancer [45]. Inadequate knowledge, language barriers and unfamiliarity with primary healthcare screening are the main reasons for this disparity [11, 29]. Community navigators haven been employed in the United States to educate immigrant minorities about cancer and direct them to primary care facilities for screening [46]. In two studies, cancer knowledge and adherence with screening recommendation for breast and cervical cancer significantly increased among Hispanic women after culturally sensitive community navigators supported them [29, 47]. When physicians referred patients for mammogram or colposcopy, bilingual navigators investigated barriers to seeking primary care, suggested possible solutions, coached patients about breast or cervical cancer,  its prevention, the importance of screening, and aided with transportation, childcare and scheduling appointments. These activities led to greater participation in primary screening [29, 47]. Similar success was also observed when navigators educated Hispanic and African American patients for colon cancer screening in primary care [11]. In a church-based community navigator study, non-significant increases in cancer screening participation were found [46].

### Community navigator models

Although different community navigation programs were described across the studies, the only established model of care used was ‘*Promotores de Salud*’ (Community Health Workers). Among the studies identified in this review, five [21, 26, 38, 39, 41] used this model, while the other studies did not report use of any specific model. In ‘*Promotores de Salud*’ model community navigators known as *promotores* were recruited from the residents of target Latino communities and were trained by a team from a community based outreach program known as ‘*Salud Para Su Corazon*’ (*SPSC*) (Health for Your Heart), which was developed by the National Heart, Lung, and Blood Institute (NHLBI) for prevention and control of cardiovascular risks among vulnerable Latino population. Subsequently the trained community navigators (*promotores*) recruited study participants from target Latino communities by direct approach, advertisement, and referral from medical professionals and randomly divided them in control and intervention groups. The participants in the intervention group received training from the navigators in accordance with the ‘heart health curriculum’ developed by NHLBI, which is a 9-week program comprising different educational approaches to train Latinos for cardiovascular disease management [21, 26, 38, 39, 41, 48]. Study outcomes included clinical factors (BMI, weight, blood pressure, cholesterol level etc.) and lifestyle behaviors (diet, physical exercise, smoking, drinking etc.). Navigators educated the intervention participants during group sessions and followed up with home visits and telephone calls. In all the studies significant improvements were observed in cardiovascular risk factors among the intervention group [21, 26, 38, 39, 41 , 48]. Although other studies did not apply a defined community navigator model like ‘*Promotores de Salud*’, the approaches used were quite similar and most of the studies reported positive outcomes after navigator intervention.

### Cost considerations

Although cost-effectiveness is a major factor for any community outreach program, none of the selected studies reported cost-effectiveness. However, we found one study in our search that reported a cost-effectiveness analysis of a community navigator intervention in Hispanic adults with type 2 diabetes [49]. We did not include this study in our results because it did not report a pre-specified outcome, and hence did not meet our inclusion criteria. The analysis was done by the University of Texas Community Outreach (UTCO) intervention, which is a community-based diabetes education and self-management program. The study population was Hispanic adults with type 2 diabetes, who were patients at the Mercy Clinic in Laredo, Texas from October 2009 to January 2010. The intervention by five state-certified community navigators and one nurse educator included classroom health education classes, nutrition classes, exercise classes, home visits, and counseling sessions. To estimate the long-term cost-effectiveness the authors forecasted disease outcomes, quality-adjusted life years (QALYs) gained, and lifetime costs associated with attaining different HbA1c levels 20 years into the future. To assess program costs they included staff and participant time for home visits, educational classes, counseling sessions, exercise classes, class-related logistics, and mileage related to home visits. Over the 20-year time horizon, they estimated a cost-effectiveness for the program of $33,319 USD per QALY gained [49]. Interventions for diabetes control or management are considered cost-effective if they fall under the threshold of $50,000 USD per QALY gained [50].

### Scope of community navigator programs for immigrant population in Canada

Canada is at a very early stage in adopting community navigators for immigrant populations, which is reflected by the fact that only one Canadian study qualified for the review [20]. However, findings from this study revealed that the MCHB model was a successful alliance between community-based organizations and the public healthcare system targeting barriers to accessing primary care for a vulnerable immigrant population. We found one additional study that demonstrated the feasibility of a peer leader training program to provide diabetes self-management support to a South Asian community in Vancouver [51], however, as it did not evaluate outcomes of interest, it was not included in the review.

According to Canadian Partnership Against Cancer (CPAC), the application of navigators in cancer care has achieved multifaceted benefits for patients, healthcare providers and the system [6, 52]. They have helped improve patients’ understanding of disease, preparation for and compliance with treatments, coping skills, and access to healthcare services [6, 52]. From health providers’ perspective, community navigators improved their interest in collaboration and teamwork, workplace satisfaction, and satisfaction with care provided. The advantages for the healthcare system included enhanced patient satisfaction, better management of oncological emergencies, improved coordination between hospital and community-based services, reduction of service duplication, potential improvement in continuity of care, improvement in quality and consistency of community cancer care [6, 52]. The positive outcomes of community navigator intervention in Canadian cancer care suggests that this approach would also help reducing the health disparities among the countrys' vulnerable immigrant population. 

Outcomes from the majority of the studies we reviewed suggest that there is an opportunity for Canada to address healthcare gaps for immigrant and ethnic minorities as well as the general population by introducing community navigator programs. The navigators should be recruited from the target community to ensure they are culturally and linguistically sensitive to the community and well accepted by the community members. It is crucial to provide rigorous training by healthcare professionals so that navigators are well prepared to educate the target population in chronic disease management, and support them in navigating the primary healthcare system.

### Limitations

The small number of studies in immigrant populations limited our review. Only three articles were found that studied immigrant populations specifically. All other studies were about ethnic or racial minority groups, which may have included new immigrants, but the researchers did not distinguish recently immigrated participants from others. While some of the findings of the studies among ethnic minorities may be generalizable to immigrant populations, there are differences in the challenges faced between these groups.

## Conclusion

Community navigators have served as educators and conducted outreach programs in order to improve the health disparities among immigrant and ethnic minorities in the United States. In all but four [32–34, 46] of the studies discussed above, navigator interventions significantly improved the primary outcomes related to chronic disease management and barriers to accessing primary healthcare for screening. Immigrant and ethnic communities make up a large portion of the population in Canada and the United States [14]. Canada may have a universal healthcare system, but language, cultural and religious limitations among immigrant populations keep many immigrants from accessing primary healthcare [16, 17, 53]. Healthcare authorities have tried to address the language barrier between immigrant patients and physicians by using professional translators, but the attempt was not very effective due to medical incompetence of the translator and patient confidentiality issues [54, 55]. Similarly, providing educational literature in different languages has not been very beneficial as printed educational materials alone are not enough to overcome cultural barriers [56]. Based on evidence in this review, culturally competent guidance provided by navigators from a patient’s own ethnic community might play a major role in overcoming barriers to healthcare. As immigrants are becoming an increasing proportion of the United States and Canadian populations the potential utility of community navigators for these groups may also grow. However, more effective research that focuses specifically upon immigrant populations will be necessary to determine the medical, social, and economic benefits of community navigators.

## References

[1] Albarran CR, Heilemann MV, Koniak-Griffin D (2014). Promotoras as facilitators of change: Latinas’ perspectives after participating in a lifestyle behaviour intervention program. J Adv Nurs.

[2] Balcazar H, Alvarado M, Ortiz G (2011). Salud para su corazon (Health for your heart) community health worker model: Community and clinical approaches for addressing cardiovascular disease risk reduction in hispanics/latinos. J Ambul Care Manage.

[3] Hilfinger Messias DK, Parra-Medina D, Sharpe PA, Treviño L, Koskan AM, Morales-Campos D (2013). Promotoras de Salud: Roles, Responsibilities, and Contributions in a Multi-Site Community-Based Randomized Controlled Trial. Hisp Health Care Int.

[4] Hurtado M, Spinner JR, Yang M, Evensen C, Windham A, Ortiz G, et al. Knowledge and behavioral effects in cardiovascular health: Community Health Worker Health Disparities Initiative, 2007–2010. Preventing Chronic Disease: Public Health Research, Practice, and Policy. 2014;11 Feb:Art 130250–9. 10.5888/pcd11.130250 PMC3929339 24524426

[5] Johnson CM, Sharkey JR, Dean WR, St John JA, Castillo M (2013). Promotoras as research partners to engage health disparity communities. J Acad Nutr Diet.

[6] Foundation TC (2013). Health System Navigators: Band-Aid or Cure?.

[7] Walkinshaw E (2011). Patient navigators becoming the norm in Canada. Can Med Assoc J.

[8] Thompson JR, Horton C, Flores C. Advancing diabetes self-management in the Mexican American population: A community health worker model in a primary care setting. Diabetes Educ. 2007;33 Suppl 6:159S–65. 10.1177/0145721707304077 17620396

[9] Ahmed S, Shommu NS, Rumana N, Barron GR, Wicklum S, Turin TC. Barriers to Access of Primary Healthcare by Immigrant Populations in Canada: A Literature Review. J Immigr Minor Health. 2015 Sep 12. PubMed Epub 2015/09/14. Eng. 10.1007/s10903-015-0276-z 26364053

[10] Staten LK, Cutshaw CA, Davidson C, Reinschmidt K, Stewart R, Roe DJ (2012). Effectiveness of the Pasos Adelante chronic disease prevention and control program in a US-Mexico border community, 2005–2008. Prev Chronic Dis.

[11] Chen LA, Santos S, Jandorf L, Christie J, Castillo A, Winkel G (2008). A Program to Enhance Completion of Screening Colonoscopy Among Urban Minorities. Clin Gastroenterol Hepatol.

[12] Immigration and Ethnocultural Diversity in Canada, 2011. Statistics Canada. Retrieved on March 26, 2015 from (http://www12.statcan.gc.ca/nhs-enm/2011/as-sa/99-010-x/99-010-x2011001-eng.cfm).

[13] Racial & Ethnic Minority Populations, 2010. Centers for Disease Control and Prevention. Retrieved March 26, 2015 from (http://www.cdc.gov/minorityhealth/populations/remp.html).

[14] United Nations Department of Economic and Social Affairs Population Division. World Migration in Figures. 2013, 3–4 October. Retrieved March 29, 2015 from (http://www.oecd.org/els/mig/World-Migration-in-Figures.pdf).

[15] Kelly E, Ivers N, Zawi R, Barnieh L, Manns B, Lorenzetti DL (2015). Patient navigators for people with chronic disease: protocol for a systematic review and meta-analysis. Syst Rev.

[16] Pottie KMDM, Ng EP, Spitzer DP, Mohammed AM, Glazier RMDMPH (2008). Language Proficiency, Gender and Self-reported Health: An Analysis of the First Two Waves of the Longitudinal Survey of Immigrants to Canada. Can J Public Health.

[17] Beiser M (2005). The health of immigrants and refugees in Canada. Can J Public Health.

[18] Arksey H, O’Malley L (2005). Scoping studies: towards a methodological framework. Int J Soc Res Methodol.

[19] Moher D, Liberati A, Tetzlaff J, Altman DG (2009). Preferred reporting items for systematic reviews and meta-analyses: the PRISMA statement. J Clin Epidemiol.

[20] Ospina ST. Uncovering the Role of Community Health Worker/Lay Health Worker Programs in Addressing Health Equity for Immigrant and Refugee Women in Canada: An Instrumental and Embedded Qualitative Case Study: University of Ottawa; 2013.

[21] Balcazar H, Alvarado M, Hollen ML, Gonzalez-Cruz Y, Pedregon V (2005). Evaluation of Salud Para Su Corazon (Health for your Heart) -- National Council of La Raza Promotora Outreach Program. Prev Chronic Dis.

[22] Lujan J, Ostwald SK, Ortiz M (2007). Promotora diabetes intervention for Mexican Americans. Diabetes Educ.

[23] Islam NS, Zanowiak JM, Wyatt LC, Kavathe R, Singh H, Kwon SC (2014). Diabetes prevention in the new york city sikh asian Indian community: A pilot study. Int J Environ Res Public Health.

[24] Koniak-Griffin D, Brecht ML, Takayanagi S, Villegas J, Melendrez M, Balcazar H (2014). A community health worker-led lifestyle behavior intervention for Latina (Hispanic) women: Feasibility and outcomes of a randomized controlled trial. Int J Nurs Stud.

[25] Martin MA, Catrambone CD, Kee RA, Evans AT, Sharp LK, Lyttle C (2009). Improving asthma self-efficacy: developing and testing a pilot community-based asthma intervention for African American adults. J Allergy Clin Immunol.

[26] Balcazar H, Alvarado M, Cantu F, Pedregon V, Fulwood R (2009). A promotora de salud model for addressing cardiovascular disease risk factors in the US-Mexico border region. Prev Chronic Dis.

[27] Fedder DO, Chang RJ, Curry S, Nichols G (2003). The effectiveness of a community health worker outreach program on healthcare utilization of west Baltimore City Medicaid patients with diabetes, with or without hypertension. Ethn Dis.

[28] Schwartz R, Powell L, Keifer M. Family-based risk reduction of obesity and metabolic syndrome: An overview and outcomes of the Idaho partnership for hispanic health. J Health Care Poor Underserved. 2013;24(Suppl2):129–44. 10.1353/hpu.2013.0106 23727970

[29] Percac-Lima S, Benner CS, Lui R, Aldrich LS, Oo SA, Regan N (2013). The impact of a culturally tailored patient navigator program on cervical cancer prevention in Latina women. J Women’s Health.

[30] Spencer MS, Rosland AM, Kieffer EC, Sinco BR, Valerio M, Palmisano G (2011). Effectiveness of a community health worker intervention among African American and Latino adults with type 2 diabetes: a randomized controlled trial. Am J Public Health.

[31] Prezio EA, Cheng D, Balasubramanian BA, Shuval K, Kendzor DE, Culica D (2013). Community Diabetes Education (CoDE) for uninsured Mexican Americans: a randomized controlled trial of a culturally tailored diabetes education and management program led by a community health worker. Diabetes Res Clin Pract.

[32] Corkery E, Palmer C, Foley ME, Schechter CB, Frisher L, Roman SH (1997). Effect of a bicultural community health worker on completion of diabetes education in a Hispanic population. Diabetes Care.

[33] Palmas W, Findley SE, Mejia M, Batista M, Teresi J, Kong J (2014). Results of the Northern Manhattan diabetes community outreach project: A randomized trial studying a community health horker intervention to improve diabetes care in hispanic adults. Diabetes Care.

[34] Islam N, Zanowiak J, Wyatt L, Chun K, Lee L, Kwon S (2013). A Randomized-Controlled, Pilot Intervention on Diabetes Prevention and Healthy Lifestyles in the New York City Korean Community. J Community Health.

[35] Rothschild SK, Martin MA, Swider SM, Lynas CMT, Janssen I, Avery EF (2014). Mexican American Trial of Community Health Workers: A Randomized Controlled Trial of a Community Health Worker Intervention for Mexican Americans With Type 2 Diabetes Mellitus. Am J Public Health.

[36] Perez-Escamilla R, Damio G, Chhabra J, Fernandez ML, Segura-Perez S, Vega-Lopez S (2015). Impact of a community health workers-led structured program on blood glucose control among latinos with type 2 diabetes: the DIALBEST trial. Diabetes Care.

[37] Ingram M, Torres E, Redondo F, Bradford G, Wang C, O’Toole ML (2007). The Impact of Promotoras on Social Support and Glycemic Control Among Members of a Farmworker Community on the US-Mexico Border. Diabetes Educ.

[38] Balcazar HG, Byrd TL, Ortiz M, Tondapu SR, Chavez M (2009). A randomized community intervention to improve hypertension control among mexican americans: using the promotoras de salud community outreach model. J Health Care Poor Underserved.

[39] de Heer HD, Balcazar HG, Castro F, Schulz L (2012). A path analysis of a randomized promotora de salud cardiovascular disease-prevention trial among at-risk hispanic adults. Health Educ Behav.

[40] Spinner JR, Alvarado M (2012). Salud Para Su Carozon-A Latino promotora-led cardiovascular health education program. Fam Community Health.

[41] Sanchez V, Cacari Stone L, Moffett ML, Nguyen P, Muhammad M, Bruna-Lewis S (2014). Process evaluation of a promotora de salud intervention for improving hypertension outcomes for Latinos living in a rural U.S.-Mexico border region. Health Promot Pract.

[42] Kandula NR, Dave S, De Chavez PJ, Bharucha H, Patel Y, Seguil P, Kumar S, Baker DW, Spring B, Siddique J (2015). Translating a heart disease lifestyle intervention into the community: the South Asian Heart Lifestyle Intervention (SAHELI) study; a randomized control trial. BMC Public Health.

[43] Ursua RA, Aguilar DE, Wyatt LC, Katigbak C, Islam NS, Tandon SD (2014). A community health worker intervention to improve management of hypertension among Filipino Americans in New York and New Jersey: A pilot study. Ethn Dis.

[44] Hunter JB, de Zapien JG, Papenfuss M, Fernandez ML, Meister J, Giuliano AR (2004). The Impact of a Promotora on Increasing Routine Chronic Disease Prevention among Women Aged 40 and Older at the U.S.-Mexico Border. Health Educ Behav.

[45] Goel MS, Wee CC, McCarthy EP, Davis RB, Ngo-Metzger Q, Phillips RS (2003). Racial and Ethnic Disparities in Cancer Screening. J Gen Intern Med.

[46] Allen J, Pérez J, Tom L, Leyva B, Diaz D, Torres M (2014). A Pilot Test of a Church-Based Intervention to Promote Multiple Cancer-Screening Behaviors among Latinas. J Cancer Educ.

[47] Livaudais JC, Coronado GD, Espinoza N, Islas I, Ibarra G, Thompson B (2002). Educating Hispanic women about breast cancer prevention: evaluation of a home-based promotora-led intervention. J Women’s Health.

[48] Anders RL, Balcazar H, Paez L (2006). Hispanic community-based participatory research using a promotores de salud model. Hisp Healthcare Int.

[49] Brown HS, Wilson KJ, Pagan JA, Arcari CM, Martinez M (2012). Cost-effectiveness analysis of a community health worker intervention for low-income Hispanic adults with diabetes. Prev Chronic Dis.

[50] Li R, Zhang P, Barker LE, Chowdhury FM, Zhang X (2010). Cost-effectiveness of interventions to prevent and control diabetes mellitus: a systematic review. Diabetes Care.

[51] Tang TS, Sohal PS, Garg AK (2013). Evaluating a diabetes self-management support peer leader training programme for the English- and Punjabi-speaking South-Asian community in Vancouver. Diabet Med.

[52] CPAC. Annual Report. Toronto: Canadian Partnership Against Cancer, 2009–10.

[53] Sanou D, O'Reilly E, Ngnie-Teta I, Batal M, Mondain N, Andrew C, Newbold BK, Bourgeault IL (2014). Acculturation and nutritional health of immigrants in Canada: a scoping review. J Immigr Minor Health.

[54] Cave A, Maharaj U, Gibson N, Jackson E (1995). Physicians and immigrant patients. Cross-cultural communication. Can Fam Physician.

[55] Papic O, Malak Z, Rosenberg E (2012). Survey of family physicians’ perspectives on management of immigrant patients: Attitudes, barriers, strategies, and training needs. Patient Educ Couns.

[56] Dastjerdi M (2012). The case of Iranian immigrants in the greater Toronto area: a qualitative study. Int J Equity Health.

